# A detection of benzimidazole resistance-associated SNPs in the isotype-1 β-tubulin gene in *Haemonchus contortus* from wild blue sheep (*Pseudois nayaur*) sympatric with sheep in Helan Mountains, China

**DOI:** 10.1186/s12917-019-1838-4

**Published:** 2019-03-12

**Authors:** Dong-dong Shen, Zhi-wei Peng, Min Hu, Zong-ze Zhang, Zhi-jun Hou, Zhen-sheng Liu

**Affiliations:** 1 College of Wildlife Resources, Northeast Forestry University, Harbin, China; 2 Key Laboratory of Wildlife Conservation, China State Forestry Administration, Harbin, China; 3 State Key Laboratory of Agricultural Microbiology, College of Veterinary Medicine, Huazhong Agricultural University, Wuhan, China

**Keywords:** Blue sheep, *Haemonchus contortus*, Isotype-1 β-tubulin gene, Benzimidazole resistance, Helan Mountains

## Abstract

**Background:**

Benzimidazole (BZ) resistance is an increasingly serious problem due to the excessive use of this anthelmintic for controlling *Haemonchus contortus*, which is one of the major gastrointestinal nematodes infecting small ruminants worldwide. Three known single nucleotide polymorphisms (SNPs), F167Y (TAC), E198A (GCA) and F200Y (TAC), in the isotype-1 β-tubulin gene of *H. contortus* are associated with BZ resistance. Comprehending the spread and origins of BZ resistance-associated SNPs has important implications for the control of this nematode.

**Results:**

Twenty-seven adult *H. contortus* were harvested from wild blue sheep (*Pseudois nayaur*), small wild ruminants sympatric with domestic ruminants, inhabiting the Helan Mountains, China, to monitor the status of BZ resistance. In addition, 20 adult *H. contortus* from domestic sheep sympatric with this wild ruminant and 36 isotype-1 β-tubulin haplotype sequences of *H. contortus* (two of these haplotypes, E198A3 and E198A4, possessed resistance-associated SNP E198A (GCA) from domestic ruminants in eight other geographical regions of China were used to further define the origins of BZ resistance-associated SNPs within the worms collected from blue sheep. The BZ resistance-associated SNP E198A was detected, whereas SNPs F167Y (TAC) and F200Y (TAC) were not found within the worms collected from blue sheep, and the frequency of homozygous resistant E198A (GCA) was 7.40%. The evolutionary tree and network showed consistent topologies for which there was no obvious boundary among the worms from the wild and domestic hosts, and two haplotypes (E198A1 and E198A2) possessing E198A from the wild blue sheep had two different independent origins. E198A1 had the same origin with E198A3 but E198A2 had a different origin with them. Population genetic analyses revealed a low level of *Fst* values (ranging from 0 to 0.19749) between all *H. contortus* worm groups in China.

**Conclusions:**

Results of the current study of the three BZ resistance-associated SNPs of *H. contortus* from wild blue sheep suggested that only E198A (GCA) was present within the worms collected from the wild ruminants and had multiple independent origins.

**Electronic supplementary material:**

The online version of this article (10.1186/s12917-019-1838-4) contains supplementary material, which is available to authorized users.

## Background

*Haemonchus contortus* is one of the main gastrointestinal nematodes that infect small ruminants worldwide [1]. A prolific breeder, one adult female worm can produce nearly ten thousand eggs every day [2]. During this worm’s parasitic period, eggs are continually expelled from the infected hosts and develop in the external environment, such as pastures to the third-stage larvae (L3 s) that can infect suitable ruminant hosts [3]. Since adults of this gastrointestinal nematode feed on the blood of hosts, the seriously infected ruminants may have some clinical presentation such as weight loss, anaemia and even death [4]. Thus, this nematode economically impacts livestock production [5].

In order to minimize the economic losses to animal husbandry caused by this nematode, benzimidazoles (BZs) have been widely used against this worm. However, the BZ resistance of *H. contortus* has become an increasingly serious problem due to excessive use of this anthelmintic [6]. Studies to date have reported that three different single nucleotide polymorphisms (SNPs) in the isotype-1 β-tubulin gene at codons 167 (T**T**C to T**A**C) [7], 198 (G**A**A to G**C**A) [8] and 200 (T**T**C to T**A**C) [9] are correlated with BZ resistance in *H. contortus*. The F200Y (TAC) which results in the replacement of phenylalanine with tyrosine seems to be the commonest SNP correlated with BZ resistance and has a high frequency in many countries [10]. Though the SNP F167Y (TAC) that results in the replacement of phenylalanine with tyrosine has also been detected to be associated with BZ resistance in many studies, it has a lower prevalence than F200Y (TAC), with an obviously restrained distribution in countries including the UK, the US, France, Canada, Argentina, and Brazil [7, 11–17]. SNP E198A (GCA), resulting in the replacement of glutamate with alanine, was also implicated in BZ resistance and has been found within two filed-derived *H. contortus* population collected from South Africa [8] and Australia [18]. More recently, the BZ resistance-associated SNPE198A (GCA) has been further examined using an in vitro selection of a *H. contortus* population containing both F200Y (TAC) and E298A (GCA) [19].

Understanding the spread and origins of BZ resistance-associated SNPs has important implications for the control of this nematode [20]. Consequently, population genetic studies of BZ resistance in *H. contortus* have been conducted in many countries. For instance, Zhang et al. [21] studied eight geographic populations of *H. contortus* in China and showed that SNPs E198A (GCA) and F200Y (TAC) had multiple independent origins, which revealed that BZ resistance associated alleles had repeatedly arisen in China.

According to a previous study [22], the wild blue sheep (*Pseudois nayaur*), a small wild ruminant sympatric with domestic ruminants, inhabiting the Helan Mountains, China, is also infected by *H. contortus*. Considering that BZ resistance has a wide distribution in China [21, 23], it is necessary to monitor BZ resistance within *H. contortus* populations from wild blue sheep. Therefore, the goals of this study were to (i) monitor the frequency on BZ resistance-associated alleles in *H. contortus* isolated from wild blue sheep and (ii) explore the origin of BZ resistance-associated SNPs in *H. contortus* populations from wild blue sheep.

## Results

### BZ resistance-associated SNP E198A (GCA), but not F167Y (TAC) and F200Y (TAC), were detected within the *H. contortus* worms isolated from wild blue sheep and domestic sheep

The partial isotype-1 β-tubulin gene sequences of all the *H. contortus* worms collected from the wild blue sheep and domestic sheep were successfully amplified using a nested PCR method. The BZ resistance-associated SNP E198A (GCA) was found within the worms collected from wild and domestic hosts, but F167Y (TAC) and F200Y (TAC) were not. As nematodes are diploid organisms, single peak represents homozygous allele and secondary peak detected means the heterozygous allele. The frequencies of heterozygous resistance at position 198 between the two worm groups from the wild and domestic hosts were 7.40 and 15.00%. Homozygous resistance at position 198 was only found in the worms collected from blue sheep, and the frequency of this genotype was 7.40%. Additionally, the frequencies of resistant allele at position 198 of the two worm groups from the wild blue sheep and domestic sheep were 11.11 and 7.50% (Table 1).

**Table 1:** Number and frequency (%) with 95% CI^a^ of individual worm genotypes with “susceptible” and “resistant” single nucleotide polymorphism (SNP) and the allele frequency (%) with 95% CI at codon E198A (GAA/GCA) associated with benzimidazole resistance in isotype-1 β-tubulin gene of *H. contortus* worms from sympatric blue sheep and sheep in Helan Mountains, China

Hosts	Number of genotype^b^ and frequency (%) with 95% CI Frequency^c^ (%) with 95% CI for codon E198A				
	Hs-198	Hr-198	Het-198	Susceptible	Resistant
Sheep	17 (85.00, 63.96~94.76)	0 (0.00, 0.00~16.11)	3 (15.00, 5.24~36.04)	(92.50, 80.14~97.42)	(7.50, 2.58~19.86)
Blue sheep	23 (85.20, 67.52~94.08)	2 (7.40, 2.06~23.37)	2 (7.40, 2.06~23.37)	(88.89, 77.81~94.81)	(11.11, 5.19~22.19)

^a^ *CI* Confidence interval

^b^ Genotype: Hs, homozygous susceptible; Hr, homozygous resistant; Het, heterozygote

^c^ The allele frequency for codon E198A (%) was calculated using as below: Susceptible of sheep = (Hs-198× 2 + Het-198)/(20 × 2). Resistant of sheep = (Hr-198× 2 + Het-198)/(20 × 2). Susceptible of blue sheep = (Hs-198× 2 + Het-198)/(27 × 2). Resistant of blue sheep = (Hr-198× 2 + Het-198)/(27 × 2)

### Sequence diversity and origins of SNP E198A (GCA) in the *H. contortus* worms from wild blue sheep

Two microliters aliquots of the genomic DNA from every worm were utilized to construct the pooled DNA samples that represented the two *H. contortus* groups collected from the wild and domestic hosts. The nested PCR product from the two pooled DNA samples was successfully amplified, cloned and sequenced to assess the genetic characterization of *H. contortus* worms collected from the wild blue sheep and domestic sheep; 27 and 20 isotype-1 β-tubulin gene sequences were obtained from the two worm groups, respectively, and 16 and 10 haplotypes, respectively, were defined in the two worm groups after sequence filtering. There were two shared haplotypes between the two worm groups, which led to a total of 24 haplotypes (GenBank accession nos. MH359364–MH359387). Among these, two haplotypes (E198A1 and E198A2) possessed BZ resistance-associated SNP E198A (GCA), and both were from the wild blue sheep *H. contortus* worms. A high level of haplotype diversity (0.952 and 0.926) was present in the two worm groups, and the nucleotide diversity was 0.04172 and 0.03925, respectively (Table 2).

**Table 2:** Genetic diversity indices of isotype-1 β-tubulin gene of *H. contortus* worms collected from sympatric blue sheep and sheep in Helan Mountains, China

Host	N	No. of sequences	h	No. of haplotypes with E198A	Hd	π
Sheep	20	20	10	0	0.926	0.03925
Blue sheep	27	27	16	2	0.952	0.04172
Total	47	47	24	2	0.952	0.04063

*Abbreviations*: N, *H. contortus* population size; h, number of haplotypes; Hd, haplotype diversity; π, nucleotide diversity

**Fig. 1:**
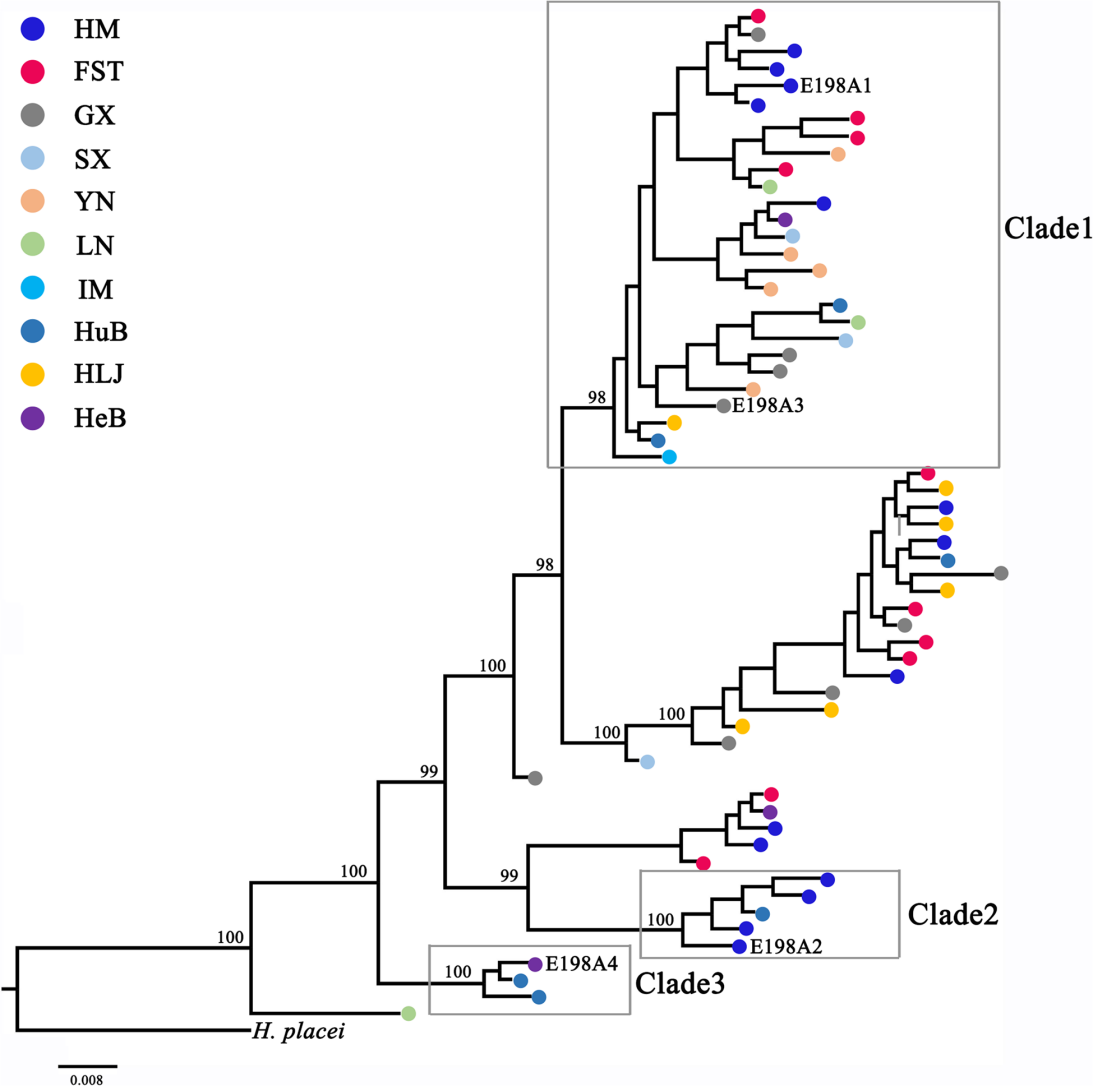
Phylogenetic tree resulting from Bayesian analysis of 60 isotype-1 β-tubulin gene sequences of *H. contortus*. The different coloured dots represent isotype-1 β-tubulin gene sequences from the different populations/sampling locations. Posterior probabilities values lower than 50 are not displayed in the tree. *Abbreviations*: FST, Farm Seven Team; GX, Guangxi; HeB, Hebei; HLJ, Heilongjiang; HM, Helan Mountains; HuB, Hubei; IM, Inner Mongolia; LN, Liaoning; SX, Shaanxi; YN, Yunnan

**Fig. 2:**
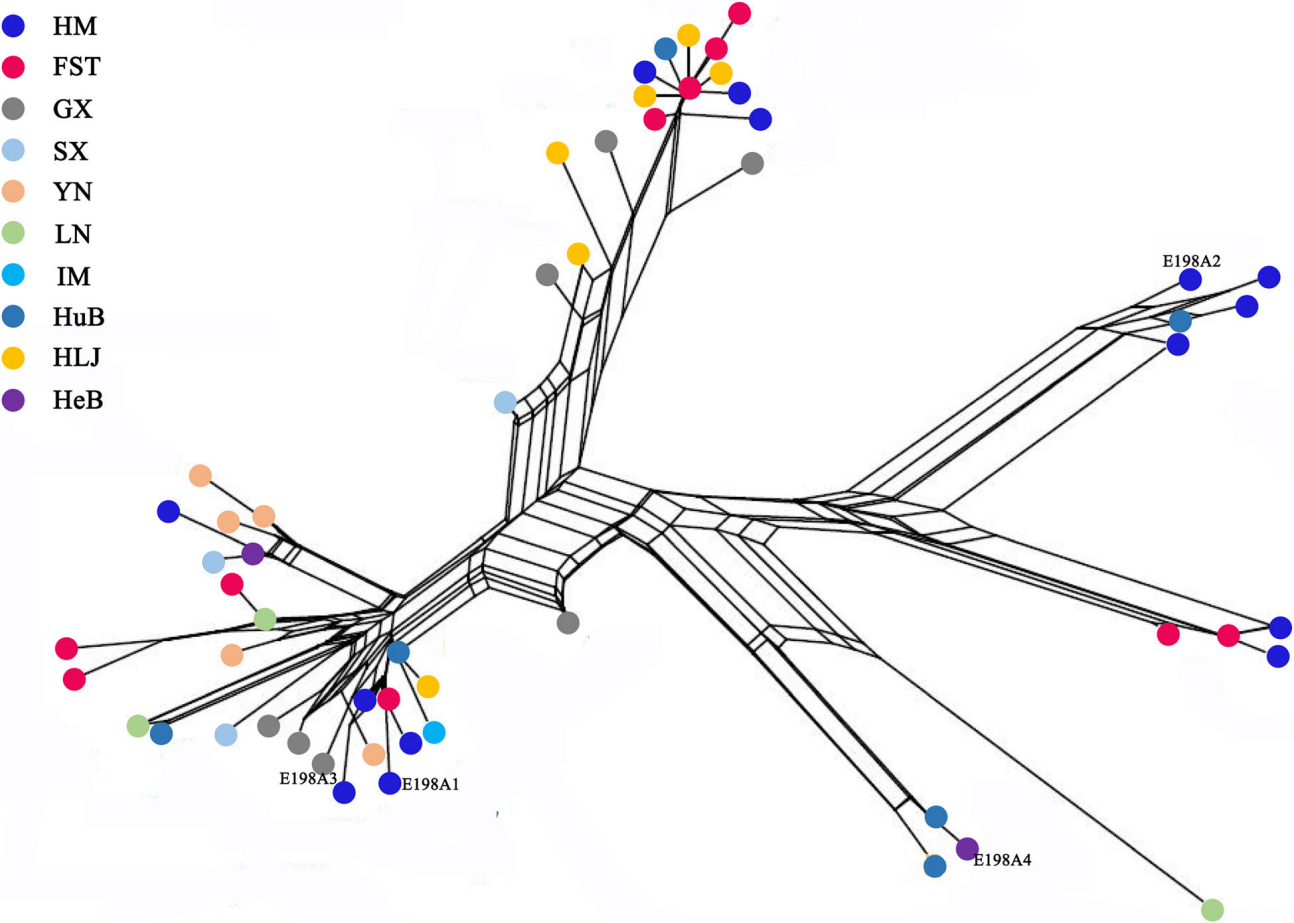
This network was built using 60 isotype-1 β-tubulin gene sequences of *H. contortus*. Isolated from wild blue sheep, sheep sympatric with the blue sheep and eight geographical regions of China. The different coloured dots represent haplotypes from the different populations/sampling locations. *Abbreviations*: FST, Farm Seven Team; GX, Guangxi; HeB, Hebei; HLJ, Heilongjiang; HM, Helan Mountains; HuB, Hubei; IM, Inner Mongolia; LN, Liaoning; SX, Shaanxi; YN, Yunnan

Another 36 isotype-1 β-tubulin haplotype sequences of *H. contortus* (two of these haplotypes, E198A3 and E198A4, possessed resistance-associated SNP E198A (GCA) from domestic ruminants in eight other geographical regions of China were used to further detect origins of the two haplotypes (E198A1 and E198A2) possessing resistance-associated SNP E198A (GCA). The evolutionary tree (Fig. 1) based on Bayesian inference (10 million generations) was hypothesized using the 60 isotype-1 β-tubulin gene haplotype sequences of *H. contortus*. And the dendrogram showed that the haplotypes from the wild and domestic hosts were randomly dispersed among the branches of the phylogenetic tree. In addition, the topology of the BI tree showed (Fig. 1) that the two haplotypes possessing resistance-associated SNP E198A (GCA) from the wild blue sheep were distributed in two distinct clades (Clade 1 and Clade 2). Moreover, Clade 2 was a distinct clade with high posterior probability (BPP = 100) composed of four HM individuals and one HuB individual. The topology of Clade 3 which was also a distinct clade with a high posterior probability (BPP = 100) composed of a resistant haplotype (E198A4) from HeB and two susceptible haplotypes from HuB was consistent with the research of Zhang et al. [21]. Corresponding with the BI tree, the topology of ML tree also showed the same result (not shown). To further test the multiple origins of the BZ resistance-associated SNP E198A (GCA) of the *H. contortus* worms from wild blue sheep, a network built by the neighbor-net method (SplitsTrees) was constructed to discern their genetic relationships. The topology (Fig. 2) revealed that there was a clear boundary between the two haplotypes (E198A1 and E198A2) possessing BZ resistance-associated SNP E198A from wild blue sheep. Additionally, to detect the genetic diversity of *H. contortus* worms isolated from the wild blue sheep and other geographical regions in China, pairwise *F*st value that is an index of genetic differentiation was calculated. A low pairwise *F*st value (0.00774) was present between the worm groups from wild blue sheep and domestic sheep in FST. Similarly, low pairwise *F*st values were also present between the wild blue sheep worm group and worm groups in other areas of China (Table 3).

**Table 3:** Pairwise *Fst* values of isotype-1 β-tubulin gene between *H. contortus* worm groups from the ten regions in China

	FST	GX	HeB	HLJ	HuB	LN	IM	SX	YN
FST	–	0.00714	0.00137	0.00767	0.01345	0.06312	0	0	**0.19064**
HM	0.00774	**0.09237**	0	0.11089	0.00616	0.09620	0	0.03547	**0.19749**

Bold indicates significant *Fst* values (*P* < 0.05)

*Abbreviations*: *FST* Farm Seven Team, *GX* Guangxi, *HeB* Hebei, *HLJ* Heilongjiang, *HM* Helan Mountains, *HuB* Hubei, *IM* Inner Mongolia, *LN* Liaoning, *SX* Shaanxi; YN, Yunnan

## Discussion

The present work is the first to monitor the three BZ resistance-associated SNPs, F167Y (TAC), E198A (GCA) and F200Y (TAC), in the isotype-1 β-tubulin gene in *H. contortus* worms from the wild blue sheep inhabiting the Helan Mountains. The BZ resistance-associated SNP E198A (GCA) was detected, whereas the other two SNPs, F167Y (TAC) and F200Y (TAC) were not found in the worms collected from wild blue sheep. In addition, for the *H. contortus* worms from the domestic sheep sympatric with the blue sheep, only heterozygosity at position 198 was detected (Table 1). It was shown that SNP E198A was dominant among the three BZ resistance-associated resistant SNPs in the two examined worm groups, which agreed with the argument proposed by other studies that SNP E198A (GCA) was most frequently encountered in China [21, 24]. The allele frequency of resistant at position 198 for the *H. contortus* worms from blue sheep was 11.11% (Table 1), which was below the average frequency (27.75%) of resistant at position 198 for the *H. contortus* worms collected from sheep and goats in eight geographical regions of China (GX, HLJ, IM, LN, SX, SZ, YD, YN) [21].

The results of our study revealed a high degree of genetic diversity within the two examined worm groups, which agreed with a previous study based on the mitochondrial gene [22]. Haplotype diversity of the isotype-1 β-tubulin sequences of *H. contortus* from the wild blue sheep and domestic sheep was at a high level (0.952 and 0.926, respectively), which was similar to a previous study (from 0.455 to 0.939) in China [21]; similarly, the nucleotide diversity of the isotype-1 β-tubulin sequences of the two *H. contortus* groups was 0.03925 and 0.04172, respectively (Table 2); these values are consistent with earlier studies on *H. contortus* from other geographical regions, such as Brazil (0.025–0.038) [11] and China (0.018–0.039) [21].

According to the topologies of the phylogenetic tree (Fig. 1) and the network (Fig. 2), the two haplotypes (E198A1 and E198A2) possessing resistant SNP E198A (GCA) from the wild blue sheep were distributed in two different groups and coupled to at least three susceptible haplotypes from the same worm group; this provided strong evidence that there were at least two independent origins of BZ resistance-associated E198A (GCA) within the *H. contortus* worms collected from the wild blue sheep. Resistant haplotype sequences E198A1 and E198A3 were contained in Clade 1 (Fig. 1), a distinct clade with high posterior probability (BPP = 98). In contrast, the supported nodes of the subclades within Clade 1 were at a low level. The sequence similarity between E198A1 and E198A3 was 98.7%. Hence, we hypothesized that one of the haplotypes (E198A1) possessing resistance-associated SNP E198A (GCA) from the wild blue sheep might have the same origin as the resistant haplotype (E198A3) from GX. The network built by the neighbor-net method also validated this hypothesis. Regarding another haplotype (E198A2) possessing resistance-associated SNP E198A (GCA) from wild blue sheep, the haplotype was shown to be clustered within three individuals from the wild blue sheep and one HuB individual and formed a distinct clade (Clade 2) with high posterior probability (BPP = 100). The network (Fig. 2) also revealed the same topology. Therefore, we hypothesized that this haplotype (E198A2) possessing resistance-associated SNP E198A (GCA) from the wild blue sheep had a different origin than the other three BZ resistance-associated E198A (GCA) SNPs in China. For E198A3 and E198A4, their distribution in the topologies of the tree and network was consistent with the study by Zhang et al. [11].

The evolutionary tree (Fig. 1) using the isotype-1 β-tubulin gene of *H. contortus* showed the same result as a previous phylogenetic analysis based on mitochondrial DNA [22]: there was no obvious boundary among these *H. contortus* worms according to geographical origin and that the haplotype sequences of *H. contortus* from wild blue sheep were randomly dispersed within the main clades of the tree. Furthermore, this phenomenon was consistent with the topology of the network (Fig. 2). Additionally, low *Fst* values (Table 3) were present among all the *H. contortus* worms collected from different regions in China. All these results indicated that a high level of gene flow without obvious geographical barriers was present among all the *H. contortus* worms in China, including the *H. contortus* worms from wild blue sheep.

The BZ resistance-associated SNP E198A (GCA) was found in the blue sheep *H. contortus* worms, which was a question worthy of consideration why the *H. contortus* worms collected from wild hosts revealed presence of markers for anthelmintic resistance. The Helan Mountains is more than just a natural habitat of wild blue sheep, it was also a main pastoral area of Ningxia Province in China. Every year, approximately 100,000 sheep were observed on the mountains from June to August [25], and the BZ resistance in China has a high prevalence. In current work, a high level of gene flow was detected based on the isotype-1 β-tubulin gene among the *H. contortus* worms collected from wild and domestic hosts, which was consistent with a previous study based on mitochondrial DNA [22]. The gene flow is determined by the life histories of parasite, host movement, effective population sizes and multiple host species being reared together in common grazing pastures [26]. Therefore, we assumed that the high level of gene flow among the *H. contortus* worms from wild and domestic hosts was caused by the sympatric grazing. Consequently, BZ resistance mutations of *H. contortus* from domestic hosts were spread into this wild hosts *H. contortus* worms because of the sympatric grazing. Results of current work revealed that one of the haplotypes (E198A1) possessing resistance-associated SNP E198A (GCA) from the wild blue sheep might have the same origin as the resistant haplotype (E198A3) from GX, which validated this hypothesis.

## Conclusions

The present study is the first to monitor the three BZ resistance-associated SNPs F167Y (TAC), E198A (GCA) and F200Y (TAC) in the isotype-1 β-tubulin gene in *H. contortus* worms from the wild blue sheep inhabiting Helan Mountains. BZ resistance-associated SNP E198A (GCA) was detected and the frequency of homozygous resistant was 7.40%, whereas another two SNPs F167Y (TAC) and F200Y (TAC) were not found. Genetic relationships analysis revealed that the two haplotypes (E198A1 and E198A2) possessing resistant SNP E198A from the wild blue sheep had two different independent origins.

## Methods

### Parasite material and DNA extraction

**Fig. 3:**
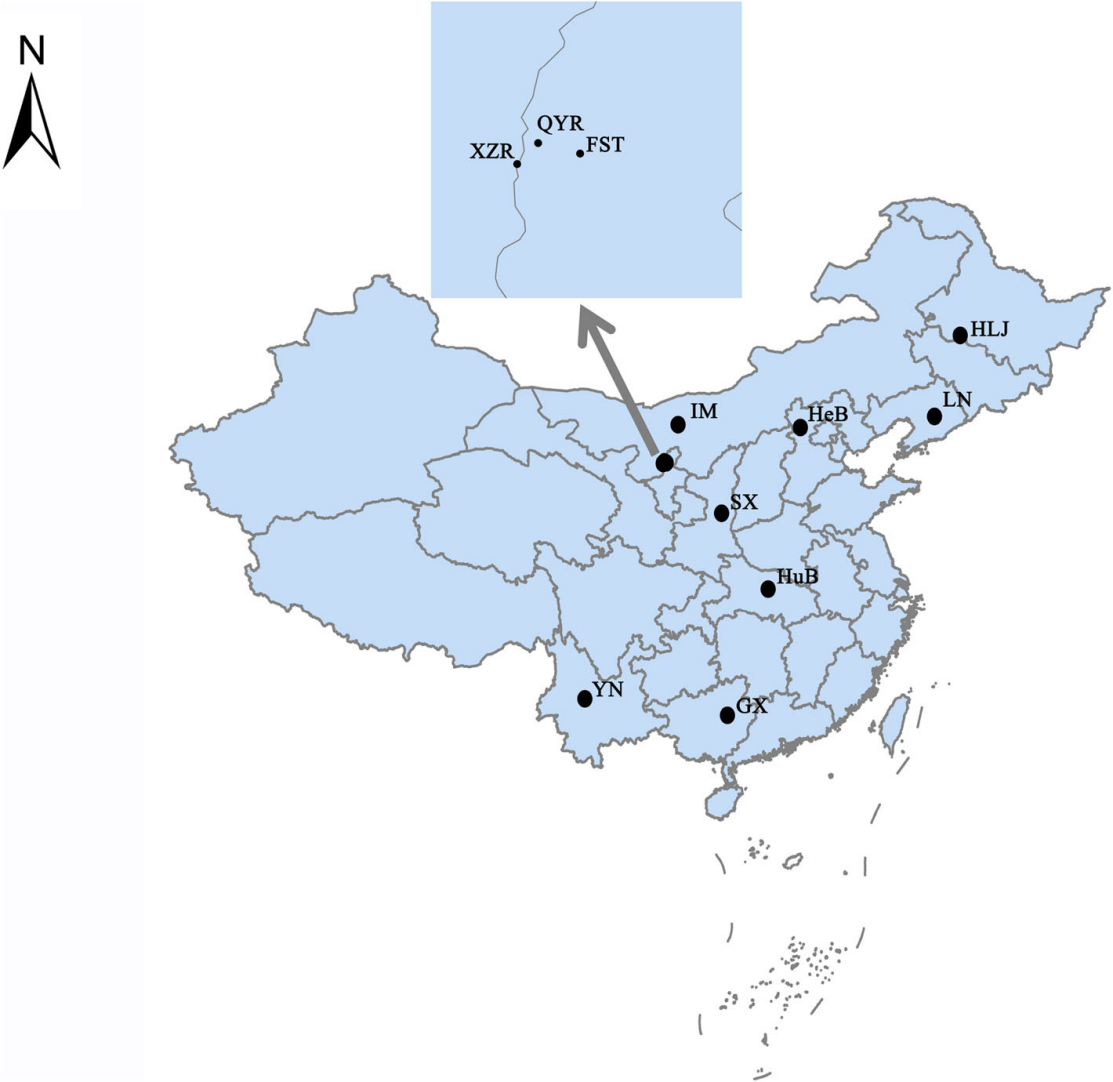
Sampling sites in this study and eight other geographical regions of China where *H. contortus* originated from the wild blue sheep and domestic ruminants. Qingyang Ravine and Xiazi Ravine belong to the Helan Mountains. *Abbreviations*: QYR, Qingyang Ravine; XZR, Xiazi Ravine; FST, Farm Seven Team; GX, Guangxi; HeB, Hebei; HLJ, Heilongjiang; HuB, Hubei; IM, Inner Mongolia; LN, Liaoning; SX, Shaanxi; YN, Yunnan

Wild blue sheep is widely distributed and has no any natural predators in the Helan Mountains. To control population sizes of the blue sheep, local government agents used the tranquilizer guns to cull a few of old and sick individuals that were eliminated naturally by blue sheep populations. In current work, approved by the local government agents, twenty-seven adult *H. contortus* were harvested from three wild blue sheep that were culled from the population by them in the areas of Qingyang Ravine and Xiazi Ravine. Another six domestic sheep that were slaughtered by herdsmen in their daily life were randomly chosen from a mountain village (Farm Seven Team) near the natural habitat of blue sheep. And we harvested twenty adult *H. contortus* from these domestic sheep with approval of the owners. All experimental designs and animals handling were approved by the Institutional Animal Care and Use Committee of Northeast Forestry University. Detailed information about the sampling sites of the hosts was shown in Fig. 3. Worm DNA was extracted using a QIAamp DNA kit (QIAamp, Hilden, Germany) following the manufacturer’s directions and then stored at − 20 °C. All worm DNA was used to assess the BZ-associated SNPs. As described by Zhang et al. [21], two pooled DNA samples representing the two *H. contortus* populations from the wild and domestic hosts were constructed to analyze the genetic diversity.

### PCR amplification and sequencing to determine the frequencies of the isotype-1 β-tubulin gene F167Y, E198A and F200Y BZ resistance-associated SNPs in *H. contortus*

A region (~ 375 bp) that included exons 4 and 5 and their intervening intron of the isotype-1 β-tubulin gene was amplified using nested PCR with newly designed primers that flanked the three BZ-associated SNPs. The primers were designed using Oligo 7 software [27] according to the complete β-tubulin sequence of *H. contortus* (GenBank accession no. X67489). Primers BR_F1 (5′-AGGGAGCCGAGCTAGTTGATA-3′) and BR_R1 (5′-AAGTGAAGACGAGGGAATGGA-3′) were used in the first-step PCR amplification, and the reactions (25 μl) included 2.5 μl of PCR buffer, 2.5 μl of dNTPs, 2 μl of MgCl_2_, 3 μl of DNA, 1 μl of each primer, 12 μl of ddH_2_O water and 1 μl of Thermo Scientific *Taq* DNA Polymerase under the following conditions: 2 min at 94 °C, 30 cycles of 40 s at 94 °C, 30 s at 54 °C, 1 min at 72 °C; and 7 min at 72 °C. Forward primer BR_F2 (5′-CTTGGAGGAGGCACTGGAT-3′) and reverse primer BR_R2 (5′-GTGAAGACGAGGGAATGGA-3′) were used for the second-step (nested) PCR amplification, and the reactions (25 μl) were performed in 2.5 μl of PCR buffer, 2.5 μl of dNTPs, 2 μl of MgCl_2_, 2 μl of first-step PCR amplification, 1 μl of each primer, 13 μl of ddH_2_O water and 1 μl of Thermo Scientific *Taq* DNA Polymerase under the following conditions: 94 °C for 2 min, 30 cycles of 30 s at 94 °C, 15 s at 55 °C, 30 s at 72 °C; and 72 °C for 7 min. Five microliters of nested PCR product was examined on a 1.0% agarose gel to verify that the product contained a single band of the appropriate size. Column-purified nested PCR products were sent to BGI (Beijing, China) for sequencing in forward and reverse directions with the primers BR_F2 and BR_R2.

To assess the relative frequencies of the BZ resistance-associated SNPs, ChromasPro version 1.5 software was used to trace the amplifications and mainly focused on positions 167, 198 and 200 according to the threshold described by Kotze et al. [19]. Relative allelic frequencies and genotypic frequencies were calculated according to Tiwari et al. [28] and the 95% confidence intervals of these frequencies were estimated as Wilson-Score intervals using the binom-wilson tool from the epitools package in the statistical programming language R.

### Amplification, cloning and sequencingof isotype-1 β-tubulin gene to detect the BZ resistance-associated SNPs F167Y, E198A and F200Y

A partial fragment of the isotype-1 β-tubulin gene was amplified from the two pooled DNA samples, and the nested PCR products were purified and then cloned into *Escherichia coli* DH5α using the pMD19-T Easy Vector System (TaKaRa) following the manufacturer’s protocols. Twenty and 27 clones were selected from the *H. contortus* worms from domestic sheep and wild blue sheep, respectively, and sequenced in both directions (forward and reverse). The published isotype-1 β-tubulin gene (GenBank accession no. X67489) was used as the reference sequence to detect the SNPs F167Y, E198A and F200Y.

### Sequence diversity and phylogenetic analyses

All the raw sequences (isotype-1 β-tubulin gene) were aligned using ClustalX v2.0 software [29]. The haplotypes were produced using the DnaSP 5.10 software [30]; in addition, haplotype diversity and nucleotide diversity were also calculated through this software. To define the spread and origins of BZ resistance-associated SNPs in *H. contortus* from wild blue sheep, another 36 isotype-1 β-tubulin gene haplotype sequences of *H. contortus* (Additional file 1) isolated from sheep and goats in eight geographical regions of China (Fig. 1) were retrieved from the NCBI nucleotide database (https://www.ncbi.nlm.nih.gov/) to construct phylogenetic trees. Phylogenetic analyses were hypothesized using maximum likelihood (ML) and Bayesian inference (BI). The partial β-tubulin gene of *Haemonchus placei* (GenBank accession no. KJ598498) acted as the outgroup. For all the isotype-1 β-tubulin gene sequences, multiple alignments were produced using ClustalX v2.0 software [29], and ambiguously aligned regions were excluded using Gblocks-0.91 [31]. The program Modeltest 3.7 [32] was used to select the appropriate model of nucleotide substitution according to Akaike’s information criterion (AIC). ML analyses were conducted using raxmlGUI v1.5 [33], and the best-fitting nucleotide substitution model was GTR + I + G. Bootstrap branch support values (MLBS) were obtained with 1000 rapid bootstrap inferences and subsequently sought in a thorough ML search of the data set. BI analyses were hypothesized using MrBayes v3.2 [34], and the best-fitting models for the partial isotype-1 β-tubulin sequence evolution was GTR + I + G. The following parameters were applied: four Markov chain Monte Carlo (MCMC) were run for 2 runs from random starting trees for 10 million generations, and trees were sampled every 100 generations; 25,000 generations were discarded as “burn-in”, and the remaining samples were used to calculate Bayesian posterior probabilities (BPP). Topologies of phylogenetic trees were drawn using FigTree v1.4.2 software (http://tree.bio.ed.ac.uk/software/figtree). For population genetic analysis, the program Arlequin 3.5 [35] was used to calculate the pairwise *Fst* value. In addition, network based on genetic distance were generated using the neighbor-net method in the program SplitsTrees v 4.0 [36].

## Additional files

Additional file 1: **Table S1.** Information on 24 different isotype-1 β-tubulin gene haplotypes from two *H. contortus* groups isolated form sympatric blue sheep and sheep. (DOC 13 kb)

Additional file 2: **Table S2.** Information on 36 different isotype-1 β-tubulin gene haplotypes from eight *H. contortus* groups in China. (DOC 14 kb)

## Abbreviations

AIC: Akaike’s information criterion
BI: Bayesian inference
BPP: Bayesian posterior probability
BZs: Benzimidazoles
CI: Confidence interval
FST: Farm Seven Team
GX: Guangxi
HLJ: Heilongjiang
HMB: Wild blue sheep inhabiting in Helan Mountains
IM: Inner Mongolia
LN: Liaoning
MJ: Median-joining
ML: Maximum likelihood
QYR: Qingyang Ravine
SX: Shaanxi
SZ: Suizhou
TBR: Tree bisection reconnection
XZR: Xiazi Ravine
YD: Yidu
YN: Yunnan
