# Supplementary material for: A detection of benzimidazole resistance-associated SNPs in the isotype-1 β-tubulin gene in Haemonchus contortus from wild blue sheep (Pseudois nayaur) sympatric with sheep in Helan Mountains, China
Source: BMC Vet Res. 2019 Mar 12;15:89. doi: 10.1186/s12917-019-1838-4 (PMC6416841; doi:10.1186/s12917-019-1838-4)
Supplement: Supplementary file 1 — Table S1. Information on 24 different isotype-1 β-tubulin gene haplotypes from two H. contortus groups isolated form sympatric blue sheep and sheep. (DOC 13 kb) [file 12917_2019_1838_MOESM1_ESM.doc]

**Table S1** Information on 24 different isotype-1 β-tubulin gene haplotypes from two *H. contortus* groups isolated form sympatric blue sheep and sheep.

| GenBank accession no. | P198 | Host |
| --- | --- | --- |
| MH359364 | E | Sheep |
| MH359365 | E | Sheep |
| MH359366 | E | Sheep |
| MH359367 | E | Sheep |
| MH359368 | E | Sheep |
| MH359369 | E | Sheep |
| MH359370 | E | Sheep |
| MH359371 | E | Sheep |
| MH359372 | E | Sheep |
| MH359373 | E | Sheep |
| MH359374 | A | Blue Sheep |
| MH359375 | E | Blue Sheep |
| MH359376 | E | Blue Sheep |
| MH359377 | E | Blue Sheep |
| MH359378 | E | Blue Sheep |
| MH359379 | E | Blue Sheep |
| MH359380 | E | Blue Sheep |
| MH359381 | E | Blue Sheep |
| MH359382 | E | Blue Sheep |
| MH359383 | E | Blue Sheep |
| MH359384 | A | Blue Sheep |
| MH359385 | E | Blue Sheep |
| MH359386 | E | Blue Sheep |
| MH359387 | E | Blue Sheep |
