# Supplementary material for: A detection of benzimidazole resistance-associated SNPs in the isotype-1 β-tubulin gene in Haemonchus contortus from wild blue sheep (Pseudois nayaur) sympatric with sheep in Helan Mountains, China
Source: BMC Vet Res. 2019 Mar 12;15:89. doi: 10.1186/s12917-019-1838-4 (PMC6416841; doi:10.1186/s12917-019-1838-4)
Supplement: Supplementary file 2 — Table S2. Information on 36 different isotype-1 β-tubulin gene haplotypes from eight H. contortus groups in China. (DOC 14 kb) [file 12917_2019_1838_MOESM2_ESM.doc]

**Table S2** Information on 36 different isotype-1 β-tubulin gene haplotypes from eight *H. contortus* groups in China.

| GenBank accession no. | P198 | Location |
| --- | --- | --- |
| KX258881 | E | GX |
| KX258883 | E | GX |
| KX258884 | E | GX |
| KX258885 | E | GX |
| KX258886 | E | GX |
| KX258887 | E | GX |
| KX258888 | E | HeB |
| KX258889 | E | HeB |
| KX258897 | E | HLJ |
| KX258898 | E | HLJ |
| KX258899 | E | HLJ |
| KX258901 | E | HLJ |
| KX258902 | E | HLJ |
| KX258903 | E | HuB |
| KX258905 | E | HuB |
| KX258907 | E | HuB |
| KX258909 | E | HuB |
| KX258910 | E | HuB |
| KX258913 | E | LN |
| KX258915 | E | IM |
| KX258916 | E | SX |
| KX258919 | E | SX |
| KX258921 | E | SX |
| KX258923 | E | YN |
| KX258924 | E | YN |
| KX258925 | E | YN |
| KX258926 | E | YN |
| KX258927 | A | GX |
| KX258930 | A | HeB |
| KX258939 | E | GX |
| KX258940 | E | GX |
| KX258941 | E | HLJ |
| KX258942 | E | HuB |
| KX258943 | E | LN |
| KX258944 | E | LN |
| KX258946 | E | YN |

*Abbreviations*: GX, Guangxi; HLJ, Heilongjiang; IM, Inner Mongolia; LN, Liaoning; SX, Shaanxi; SZ, Suizhou; YD, Yidu; YN, Yunnan.
